# Multiple ground-glass nodules after lobectomy for multiple primary lung cancer: a case report

**DOI:** 10.3389/fsurg.2026.1842030

**Published:** 2026-05-28

**Authors:** Zhipeng Song, Shaohui Huang, Ziqi Wang, Quncheng Zhang, Xiaoju Zhang

**Affiliations:** 1 Department of Respiratory and Critical Care Medicine, Xinxiang Medical University, Henan Provincial People’s Hospital, Zhengzhou, China; 2 Department of Respiratory and Critical Care Medicine, Zhengzhou University People’s Hospital, Henan Provincial People’s Hospital, Zhengzhou, China; 3 Henan International Joint Laboratory of Diagnosis and Treatment for Pulmonary Nodules, Zhengzhou, China

**Keywords:** EGFR mutation, EGFR-TKI, ground-glass nodule, multiple primary lung cancer, targeted therapy

## Abstract

**Background:**

Multiple primary lung cancer (MPLC) is classified into synchronous and metachronous types. It is clinically rare for a single patient to present with both types simultaneously. EGFR tyrosine kinase inhibitors (EGFR-TKIs) are the first-line treatment for EGFR-sensitive mutant lung cancer, but their therapeutic value in unresectable GGN-type MPLC requires further validation.

**Case presentation:**

A female with no smoking history or family history of tumors underwent examination in March 2020, which revealed multiple ground-glass nodules (GGNs) in the left upper lobe. After ruling out distant metastasis, she underwent left upper lobectomy. Postoperative pathology confirmed synchronous MPLC (sMPLC), and genetic testing identified an EGFR L858R mutation. No adjuvant therapy was administered, and regular follow-up was maintained. In April 2022, follow-up computed tomography (CT) scans detected new multiple tiny GGNs in the left lower lobe. Subsequent monitoring showed progressive nodule enlargement. In 2024, bronchoscopic biopsy was performed, and pathology confirmed minimally invasive adenocarcinoma (MIA), consistent with metachronous MPLC (mMPLC). Considering the patient's previous EGFR mutation and the unresectable nature of the lesions, EGFR-TKI targeted therapy was initiated. After treatment, the multiple GGNs in the left lower lobe gradually shrank and ultimately disappeared, achieving sustained complete remission (CR). Follow-up to date has shown no recurrence or significant adverse reactions.

**Conclusion:**

Patients with multiple GGNs may develop both synchronous and mMPLC. For unresectable GGN-type MPLC with a confirmed history of EGFR-sensitive mutations, EGFR-TKI targeted therapy demonstrates definitive efficacy and can achieve complete remission.

## 1 Introduction

Multiple primary lung cancer (MPLC) refers to two or more independent malignant tumors originating within the lungs of the same patient. Based on the timing of lesion detection, MPLC can be classified as synchronous (sMPLC) or metachronous (mMPLC) (1). In recent years, with the widespread application of high-resolution computed tomography (HRCT) and the increasing prevalence of routine screening, the detection rate of MPLC manifesting as ground-glass nodules (GGNs) has been rising annually (2). GGN-type MPLC, characterized by indolent growth, relatively low malignancy, and a propensity for multicentric origin (3), has become a research hotspot in the field of lung cancer.

Persistent or metachronous GGN lesions carry potential risks of invasive transformation and malignant progression. For patients with combined synchronous and metachronous MPLC, repeated surgical resection is often limited by poor pulmonary reserve and multiple intrapulmonary lesions, leading to prominent clinical treatment dilemmas. In clinical practice, pure sMPLC or pure mMPLC is relatively common; however, cases in which both sMPLC and mMPLC coexist in the same patient are rare, rendering the diagnostic and therapeutic process more complex. EGFR mutations represent the most common driver gene alterations in lung adenocarcinoma (4), and EGFR tyrosine kinase inhibitors (EGFR-TKIs) have become the first-line treatment for advanced lung adenocarcinoma with EGFR-sensitive mutations (5). However, for MPLC manifesting as GGNs with both synchronous and metachronous components, the therapeutic efficacy and long-term management strategies of EGFR-TKIs remain inadequately supported by clinical evidence. Clinical experience in the diagnosis and treatment of such rare cases urgently needs to be accumulated.

This article reports a case of a patient who developed new metachronous multiple ground-glass nodule-type MPLC in the left lower lobe after undergoing surgery for sMPLC in the left upper lobe. The patient harbored EGFR-sensitive mutations and achieved complete remission of all lesions following EGFR-TKI targeted therapy. By reviewing the diagnostic and therapeutic process, this paper discusses the key points of differential diagnosis, the value of targeted therapy, and long-term management strategies for this dual-type MPLC, aiming to provide a reference for the clinical management of similar rare cases.

## 2 Patient information and medical history

**Figure 1:**
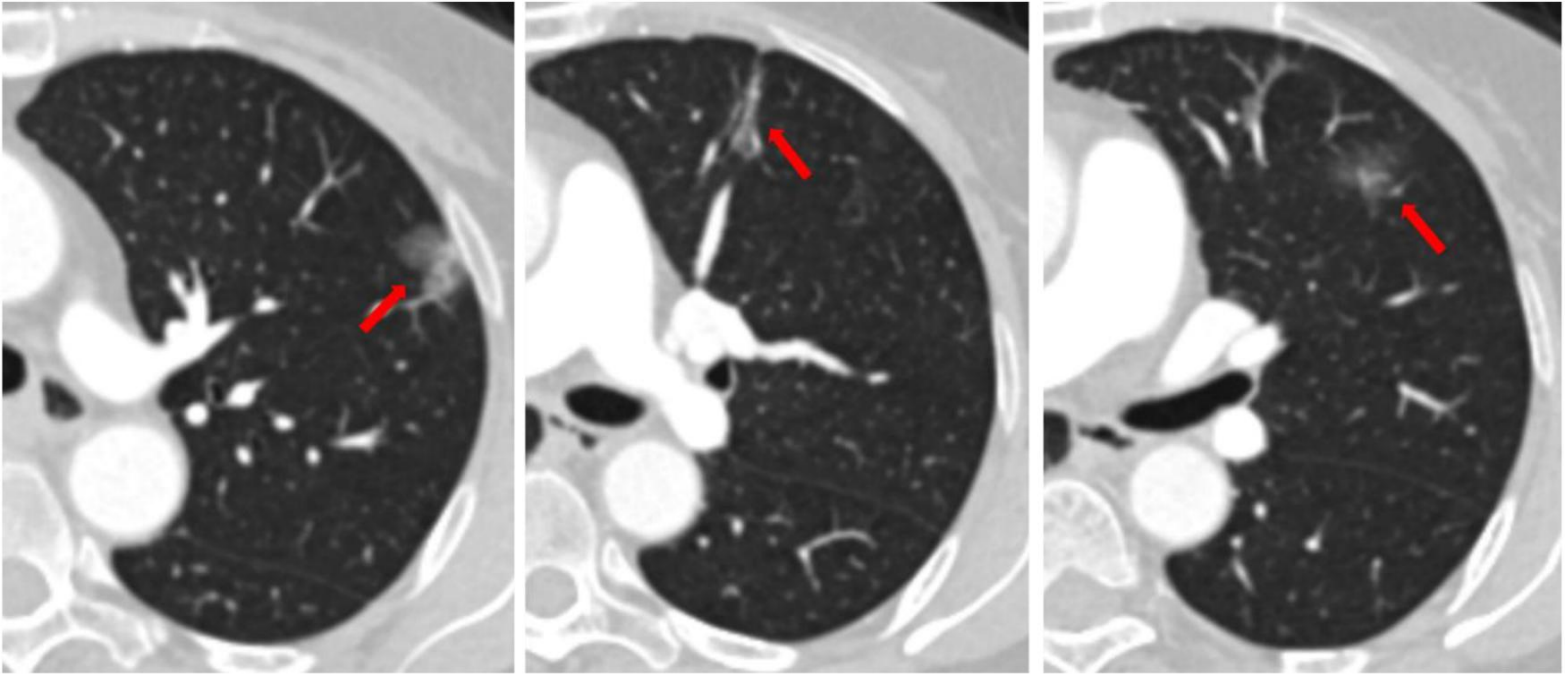
Three GGNs are visible in the left upper lobe. Postoperative pathology, from left to right, revealed IAC, IAC, and MIA, the first nodule underwent genetic testing, confirming an EGFR L858R mutation. Based on the Martini-Melamed diagnostic criteria, this was diagnosed as sMPLC.

The patient is a 74-year-old female with no history of smoking, no family history of tumors, and no known drug or food allergies. On March 14, 2020, a chest CT scan performed revealed multiple GGNs in the left upper lobe; no significant abnormalities were observed in the right lung. The specific imaging findings are shown in Figure 1. Among these, a partially solid GGN near the pleura in the left upper lobe was considered high-risk based on imaging characteristics. To exclude distant metastasis, the patient underwent a Positron Emission Tomography/Computed Tomography (PET/CT) scan, which showed no evidence of distant metastasis. Consequently, a left upper lobectomy with mediastinal lymph node dissection was performed. Postoperative pathology confirmed that the three GGNs in the left upper lobe were invasive adenocarcinoma (IAC) and minimally invasive adenocarcinoma (MIA). Based on the distribution, morphology, and pathological features of the lesions, along with the Martini-Melamed diagnostic criteria, the diagnosis was sMPLC. Genetic testing of the resected specimen revealed an EGFR L858R mutation. The patient did not receive adjuvant radiotherapy, chemotherapy, or targeted therapy after surgery and was managed with regular outpatient follow-up.

## 3 Follow-up process

**Figure 2:**
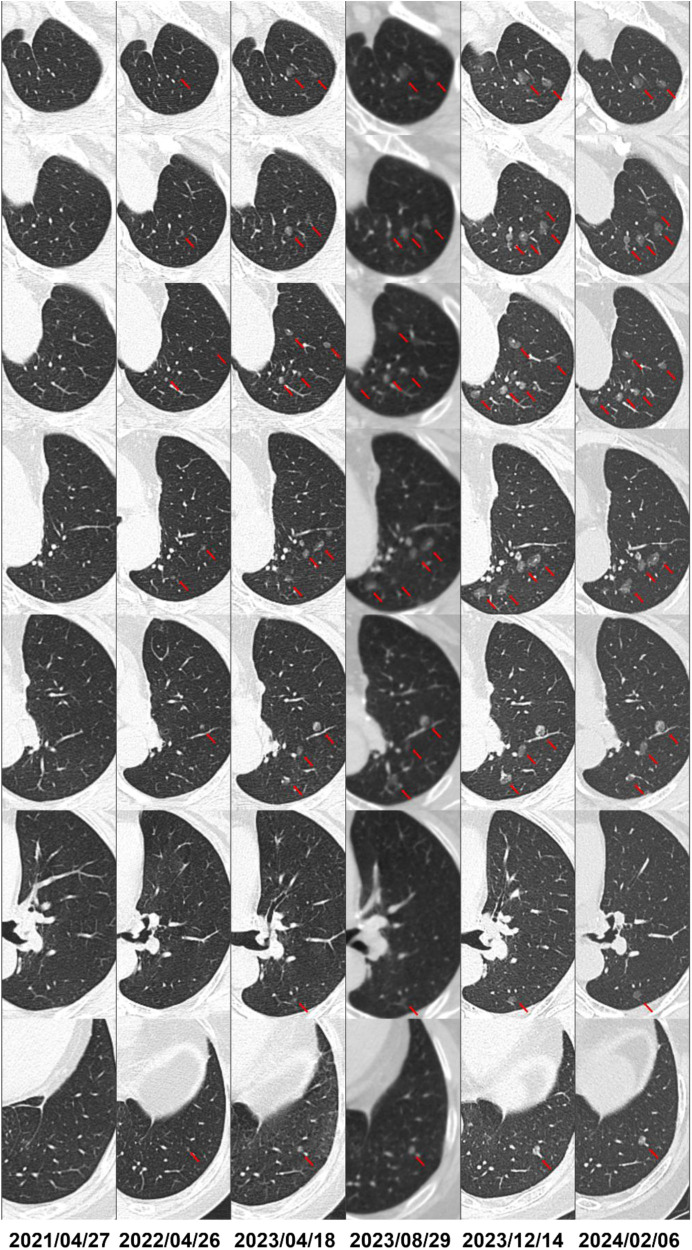
Follow-up chest CT images demonstrating the evolution of lesions in the left lower lobe. The scan on April 27, 2021, showed no abnormalities. By April 26, 2022, tiny lesions became apparent. Subsequent imaging revealed that the multiple GGNs in the left lower lobe had significantly increased in size compared to earlier examinations.

In April 2021, follow-up chest CT revealed no remarkable abnormalities. In April 2022, multiple newly emerging GGNs were detected in the left lower lobe. Considering the initial size, density and indolent growth trend of these lesions at that stage, no targeted intervention was administered, and regular imaging surveillance was maintained. Serial chest CT examinations performed in April 2023, August 2023, December 2023 and February 2024 demonstrated progressive enlargement and increased density of the multifocal GGNs in the left lower lobe. During this period, no new abnormal lesions were observed in the right lung. In terms of imaging scanning parameters: the chest CT obtained in April 2021 adopted a slice thickness of 2.0 mm, while all subsequent CT scans were performed with a slice thickness less than 1.5 mm. Unified scanning protocols were applied for all examinations, with a tube voltage of 120 kV, tube current ranging from 100 to 200 mAs, and standard chest CT reconstruction algorithms and window settings. Detailed sequential imaging manifestations are presented in Figure 2.

## 4 Physical examination

General physical examination, including assessments of the cardiovascular, respiratory, abdominal, neurological, and other systems, revealed no significant abnormalities.

## 5 Diagnostic evaluation and treatment

Laboratory tests showed no significant abnormalities in the seven tumor-associated autoantibodies or tumor markers (including CEA, CYFRA21-1, NSE, and SCC). Immunological function tests revealed a slight increase in the serum NK cell percentage and a mild decrease in CD4 + T lymphocyte count compared to previous results, while all other immune parameters were within normal limits. Ultrasonography demonstrated no enlargement or abnormalities in the superficial or axillary lymph nodes. Brain MRI and whole-body bone scan excluded the possibility of distant metastasis.

**Figure 3:**
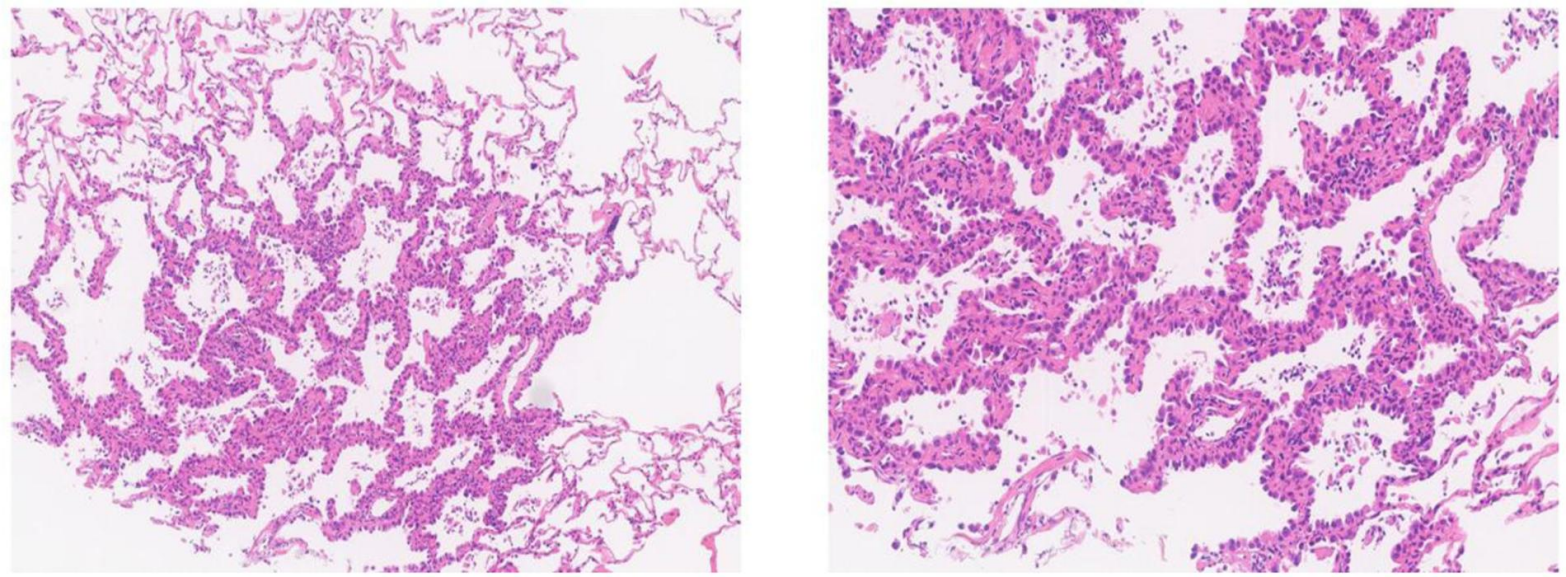
The GGN in the left lower lobe underwent bronchoscopic frozen biopsy, and the final pathological diagnosis was MIA. Based on the Martini-Melamed diagnostic criteria, this case is consistent with mMPLC.

To clarify the nature of the newly developed nodules in the left lower lobe and accurately differentiate them from intrapulmonary metastasis (IPM), we initially arranged for the patient to undergo bronchoscopic lung tissue biopsy. Postoperative pathological findings confirmed that the new lesion was at least MIA, which provided the first key evidence supporting the diagnosis of a new primary lesion rather than metastasis. See Figure 3 for details.

Subsequently, we intended to proceed with the second step of molecular characteristic analysis to compare the gene mutation profiles between the new lesion and the previously resected lesion. Regrettably, due to the extremely limited tissue sample obtained from the bronchoscopic biopsy, we were unable to perform this molecular comparison, which we fully acknowledged as a limitation in the diagnostic process.

Despite the lack of molecular evidence, we did not rush to make a diagnosis; instead, we comprehensively integrated all available clinical information—including the patient's medical history, imaging findings, and the newly obtained pathological results, fully weighing the possibility of mMPLC vs. IPM. Considering that the time interval between the initial lesion and the new lesion was more than 2 years, combined with the independent biological behavior of the new lesion, we finally concluded that the overall findings were consistent with mMPLC.

After confirming the diagnosis, we further evaluated the patient's treatment feasibility: given the patient's prior left upper lobe resection and the wide distribution of multiple nodules in the left lower lobe, further surgical resection was deemed clinically unfeasible. Taking into account the EGFR L858R sensitive mutation identified in the genetic testing of the previously resected lesion, we comprehensively judged that EGFR-TKI targeted therapy would be the most appropriate treatment strategy for the patient, and thus initiated targeted therapy to achieve comprehensive tumor control.

## 6 Pharmacological treatment

**Figure 4:**
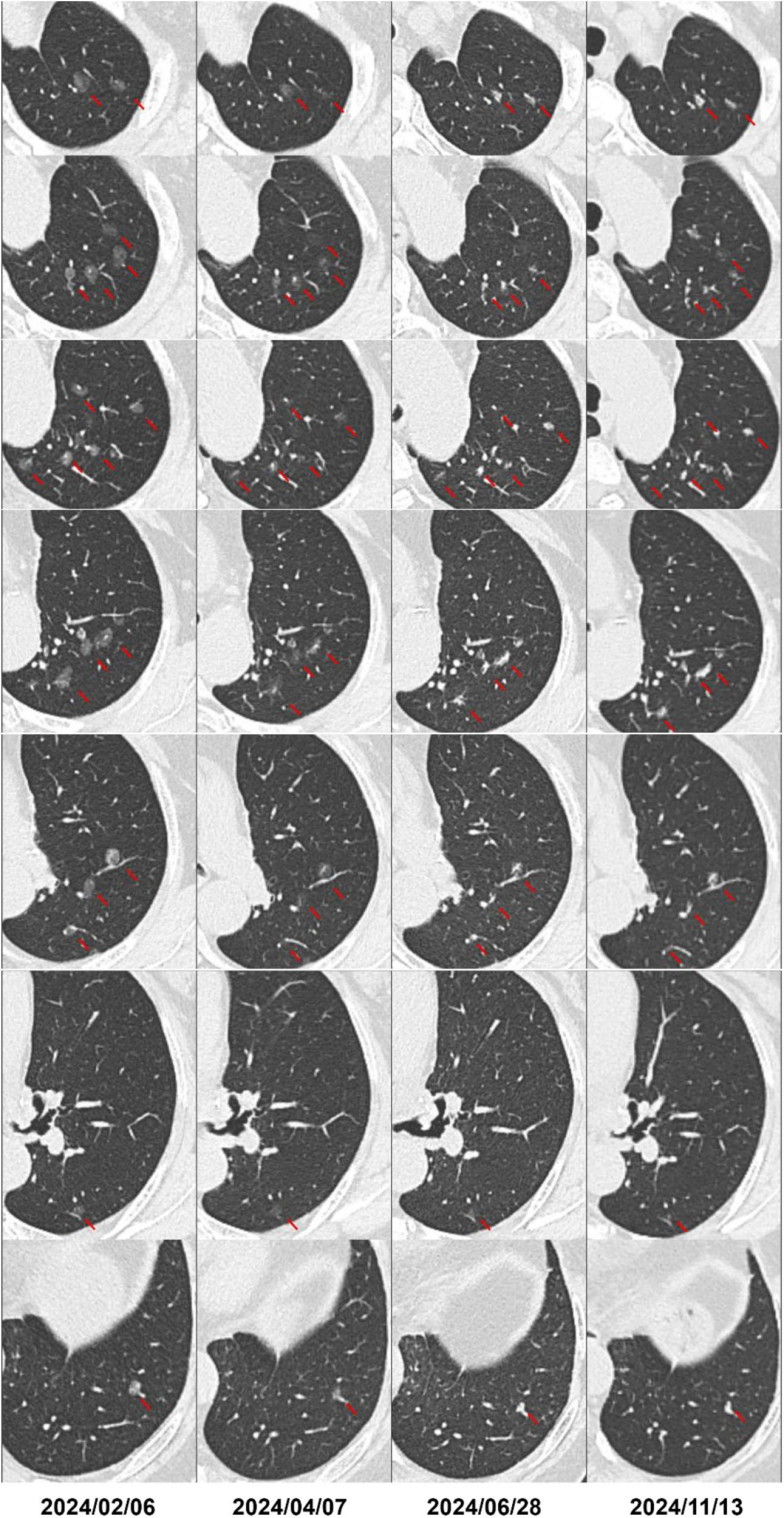
Following administration of EGFR-TKI, the multiple GGNs in the left lower lobe demonstrated a gradual decrease in size and density over time, eventually manifesting as a solid scar on chest CT.

After discharge, the patient strictly adhered to the prescribed regimen of regular oral EGFR-TKI targeted therapy and returned to the hospital for scheduled follow-up examinations. In the first month after discharge, the patient was treated with Osimertinib, 80 mg/Qd; subsequently, due to economic reasons, the treatment was switched to oral Almonertinib, 110 mg/Qd, which has been continued to date. To date, no targeted therapy-related common adverse events occurred during the treatment period. Chest CT scans performed in April 2024, June 2024, and December 2024 all indicated that the multiple ground-glass nodules in the left lower lobe progressively diminished compared to previous assessments, demonstrating a significant therapeutic response to the targeted therapy. The specific imaging findings are shown in Figure 4. To date, the patient remains under regular follow-up observation with no evidence of nodule recurrence, enlargement, or distant metastasis, and the condition continues to be stable.

## 7 Discussion

The clinical detection rate of MPLC has been rising annually with the widespread application of high-resolution chest CT screening. However, its clinical phenotypes are complex and diverse. Cases in which both synchronous and metachronous MPLC coexist in the same patient are particularly rare, and the diagnostic and therapeutic process is especially distinctive and valuable for reference, particularly in low-risk elderly female patients with no smoking history or family history of tumors.

The diagnosis of sMPLC and mMPLC centers on confirming the independent origin of multiple lesions while strictly excluding local recurrence and intrapulmonary metastasis. This requires a multidimensional comprehensive assessment integrating clinical, imaging, pathological, and systemic evaluations. Traditional diagnosis relies mainly on imaging features, histopathological morphology, and clinical time intervals. However, with the deepening understanding of tumor heterogeneity, these macroscopic or morphology-based determination methods have shown limitations in some cases. Especially when multiple nodules exhibit similar histological types, relying solely on morphological features often makes reliable differentiation difficult (6). In recent years, advances in molecular profiling and high-throughput sequencing technologies have provided new evidence for the precise diagnosis of MPLC (7). Studies have shown that differences in driver gene mutation profiles, copy number variations (CNVs), and evolutionary branch structures among different lesions can serve as important molecular evidence for determining multiple primary origins (8). In contrast, metastatic lesions derived from the same clone dissemination typically exhibit highly consistent mutation characteristics and clonal evolutionary trajectories. Therefore, integrated analysis based on multi-omics data has gradually become an important supplement to MPLC diagnosis, helping to improve diagnostic accuracy and guide individualized treatment decisions (9). Nevertheless, the current diagnostic criteria for MPLC are not fully unified, and differences in determination strategies and threshold settings exist among different studies. Future large-scale, multicenter studies are still needed to further verify the stability and clinical applicability of molecular diagnostic indicators.

GGNs are a common imaging manifestation in MPLC (10), exhibiting biological behaviors significantly distinct from traditional solid nodular lung cancer. Previous studies have indicated that GGN-type lung cancer often originates from multicentric atypical hyperplasia of alveolar epithelial cells, generally characterized by low invasiveness, slow progression, and a long tumor doubling time (11). At the pathological level, the progression typically follows a continuous spectrum from atypical adenomatous hyperplasia (AAH) to adenocarcinoma *in situ* (AIS), then to MIA, and ultimately to IAC (12), reflecting a relatively well-defined stepwise evolutionary pathway. In the clinical management of MPLC, the dynamic evolution of GGN lesions holds significant indicative value. Compared to rapidly progressing solid lesions, GGN-type lesions tend to remain stable or progress slowly over extended periods; however, they still carry the potential risk of transitioning to an invasive phenotype (13). Therefore, a single time-point imaging assessment is insufficient to fully capture their biological characteristics, and long-term, continuous imaging follow-up is crucial for identifying malignant progression. Existing research also notes that the 2–5 years post-surgery represent a high-risk window for the occurrence of mMPLC (14), during which close monitoring of changes in the size, density, and internal structure of residual or newly developed GGNs is essential. Given these characteristics, establishing a standardized follow-up strategy is of great clinical importance for MPLC patients primarily presenting with GGNs.

The rational application of EGFR-TKI targeted therapy is central to achieving favorable outcomes in this case. Studies indicate that the EGFR mutation rate in multiple primary lung cancer can reach up to 60%, and a single patient may harbor multiple EGFR-mutant carcinomas (15). In this case, postoperative genetic testing revealed an EGFR L858R sensitive mutation; however, genetic testing could not be performed on the newly developed nodules in the left lower lobe. Nevertheless, considering the biological characteristics of GGN-type MPLC with multicentric shared driver mutations, along with the patient's absence of distant metastasis and the GGN morphology of the nodules, it is reasonable to infer that the new nodules also carry EGFR-sensitive mutations. Moreover, clinical studies have confirmed that EGFR-TKI exerts definitive efficacy on unresected GGN lesions (16). Additionally, there is a case of postoperative MPLC with enlargement of residual GGNs that achieved near-complete remission following EGFR-TKI treatment (17). In this patient, after EGFR-TKI targeted therapy, the multiple GGNs in the left lower lobe gradually shrank and ultimately disappeared entirely; long-term follow-up has maintained a complete remission state. This further validates the significant therapeutic efficacy of EGFR-TKI for EGFR-mutant GGN-type MPLC and provides a safe, feasible, and effective treatment option for MPLC patients who are not candidates for surgical resection.

Targeted therapy serves as a core treatment option for patients with driver gene-positive MPLC. However, significant genomic heterogeneity exists among different lesions in MPLC, which poses considerable challenges to unified targeted treatment (18). Although MPLC manifesting as ground-glass nodules frequently harbors EGFR mutations, empirical single-agent TKI therapy may prove ineffective for patients with inconsistent driver alterations across multifocal lesions. Relevant multicenter cohort studies have demonstrated that, after a median follow-up of 6.7 years, most synchronous subsolid ground-glass nodules remained stable without progressive growth (19). Furthermore, emerging evidence indicates that adjuvant EGFR-TKI fails to improve long-term survival in high-risk stage IB EGFR-mutant NSCLC. For indolent subsolid lung tumors with an inherently favorable prognosis, routine adjuvant TKI brings limited clinical benefit regardless of targeted intervention (20). Similarly, a studies have confirmed that adjuvant EGFR-TKI cannot significantly prolong overall survival in pathological stage I (IA–IB) patients (21). Accordingly, a de-escalation treatment strategy is worthy of clinical consideration, especially for indolent subsolid MPLC lesions; delayed TKI administration until disease progression may be a more reasonable and individualized therapeutic choice. Furthermore, stereotactic body radiation therapy (SBRT) and local ablation represent vital alternative treatment options for inoperable MPLC patients. SBRT has been recommended by the NCCN Guidelines as a standard therapeutic modality for unresectable early-stage lung cancer, and its clinical application in MPLC is gradually expanding (22). Relevant studies have reported that patients with synchronous MPLC receiving SBRT achieve a median overall survival of 46.9 months, with 2- and 4-year survival rates of 74.7% and 49.2%, respectively. Although its efficacy remains inferior to surgical resection, SBRT serves as a reliable and effective local treatment for inoperable individuals (23). In addition, bronchoscopic radiofrequency ablation has emerged as a promising local therapeutic approach for ground-glass nodules (24). Nevertheless, long-term survival evidence supporting ablation in MPLC remains insufficient, and further randomized controlled trials are required to validate its clinical efficacy (25).

From the perspective of clinical management, this case provides several important insights. Firstly, for high-risk postoperative patients, long-term regular follow-up is crucial for detecting metachronous new lesions, particularly for GGN-type lesions, which, although slow-growing, may still progress to invasive adenocarcinoma. Secondly, this case demonstrates that EGFR-TKI therapy exhibits favorable efficacy for multiple GGN-like lung adenocarcinomas, suggesting that molecular targeted therapy can serve as an effective intervention when surgical resection is not feasible or lesions are widely distributed. Finally, this case underscores the importance of individualized management integrating dynamic imaging changes, pathological types, and molecular characteristics, offering a reference for early intervention and treatment strategies for metachronous multiple GGNs. However, this case also has certain limitations. First, genetic testing could not be performed due to the limited tissue sample obtained from bronchoscopic biopsy of the newly emerged left lower lobe nodules. Meanwhile, circulating tumor DNA (ctDNA) detection was not conducted in this case, because it requires additional out-of-pocket costs and imposes an extra economic burden on the patient. The lack of molecular results hinders the clarification of molecular heterogeneity between the secondary nodules and the initial primary lesion, as well as further analysis of the molecular features of MPLC. Second, for similar MPLC cases, long-term observation is still needed to assess the maintenance efficacy and resistance patterns of targeted therapy. Finally, as this is a single case, the related diagnostic and therapeutic conclusions require further validation through accumulation of more clinical cases.

## 8 Conclusion

This case represents a typical example of postoperative metachronous multiple GGNs. Its slow progression and favorable response to targeted drugs provide valuable clinical experience, while also reflecting the limitations of differentiating MPLC from intrapulmonary metastasis in the absence of molecular evidence. This highlights the need for future research to incorporate molecular clonal analysis and dynamic radiomics assessment to facilitate precise diagnosis and individualized treatment.

## Data Availability

The raw data cannot be fully publicly available due to patient privacy and ethical restrictions. Related analyzed and anonymized data are available upon reasonable request to the corresponding author.
